# Enhanced pro‐protein convertase subtilisin/kexin type 9 expression by C‐reactive protein through p38MAPK‐HNF1α pathway in HepG2 cells

**DOI:** 10.1111/jcmm.12931

**Published:** 2016-09-15

**Authors:** Chuan‐Jue Cui, Sha Li, Cheng‐Gang Zhu, Jing Sun, Ying Du, Yan Zhang, Na‐Qiong Wu, Yuan‐Lin Guo, Rui‐Xia Xu, Ying Gao, Jian‐Jun Li

**Affiliations:** ^1^ Division of DyslipidemiaFu Wai HospitalNational Center for Cardiovascular DiseasesChinese Academy of Medical SciencesPeking Union Medical CollegeBeijingChina

**Keywords:** C‐reactive protein, pro‐protein convertase subtilisin/kexin type 9, mitogen‐activated protein kinase

## Abstract

Plasma C‐reactive protein (CRP) concentration is associated positively with cardiovascular risk, including dyslipidemia. We suggested a regulating role of CRP on pro‐protein convertase subtilisin/kexin type 9 (PCSK9), a key regulator of low‐density lipoprotein (LDL) metabolism, and demonstrated the PCSK9 as a pathway linking CRP and LDL regulation. Firstly, experiments were carried out in the presence of human CRP on the protein and mRNA expression of PCSK9 and LDL receptor (LDLR) in human hepatoma cell line HepG2 cells. Treatment with CRP (10 μg/ml) enhanced significantly the mRNA and protein expression of PCSK9 and suppressed the expression of LDLR. Of note, a late return of LDLR mRNA levels occurred at 12 hrs, while the LDLR protein continued to decrease at 24 hrs, suggesting that the late decrease in LDLR protein levels was unlikely to be accounted for the decrease in LDL mRNA. Secondly, the role of PCSK9 in CRP‐induced LDLR decrease and the underlying pathways were investigated. As a result, the inhibition of PCSK9 expression by small interfering RNA (siRNA) returned partly the level of LDLR protein and LDL uptake during CRP treatment; CRP‐induced PCSK9 increase was inhibited by the p38MAPK inhibitor, SB203580, resulting in a significant rescue of LDLR protein expression and LDL uptake; the pathway was involved in hepatocyte nuclear factor 1α (HNF1α) but not sterol responsive element‐binding proteins (SREBPs) preceded by the phosphorylation of p38MAPK. These findings indicated that CRP increased PCSK9 expression by activating p38MAPK‐HNF1α pathway, with a certain downstream impairment in LDL metabolism in HepG2 cells.

## Introduction

Coronary artery disease (CAD) is increasingly being a leading health threat worldwide, and atherosclerosis has been identified as its main underlying cause 1. Of the various hypotheses offered to explain atherosclerosis, dyslipidemia and inflammation now appear to provide the key to this pathological process and the future events 1, 2, 3.

More recently, a novel modulator of lipid and cardiovascular health, pro‐protein convertase subtilisin kexin 9 (PCSK9), has attracted intensive attention from gene and protein 4, 5, 6. PCSK9 was found to be expressed mostly in liver hepatocytes, and the localization of its gene was linked to autosomal dominant hypercholesterolaemia (ADH) 7. Shortly thereafter, PCSK9 has emerged as a genetically validated target for regulating plasma low‐density lipoprotein (LDL) cholesterol (LDL‐C) levels *via* hepatic low‐density lipoprotein receptor (LDLR) degradation 8, 9. As a result, PCSK9 has revised the previous knowledge regarding cholesterol homeostasis and cardiovascular treatment.

Of the wide array of inflammatory markers that have been studied, C‐reactive protein (CRP) has received the most attention for its use in screening and risk reclassification of CAD 10, 11, 12. The acute‐phase protein CRP is a member of the pentaxin protein family involved in pattern recognition and innate immunity; it is synthesized primarily by the liver in response to inflammation. In addition to being an independent predictor of cardiovascular events, CRP is also closely associated with dyslipidemia 13, 14. It remains unclear, however, whether this association simply reflects the inflammatory milieu or whether it suggests a causative role of CRP in the progression of dyslipidemia and related cardiovascular disturbances.

The interplay between lipid metabolism and inflammation at multiple levels may be worthy of investigation in atherogenesis 15. Despite of the confirmed function of PCSK9 as a lipid modulator described above, the scenario of PCSK9 with inflammation is still unclear. Interestingly, there are some scattered researches including our previous studies 16, 17, which indicated the epiphenomenon. *In vivo* study from Kenneth *et al*. showed that the expression of PCSK9 could be markedly induced by a single‐dose intraperitoneal administration of lipopolysaccharide (LPS) in female C57BL/6 mice, which suggested a harmful role of PCSK9 on host defence. In other words, they demonstrated that infection and inflammation stimulated the expression of PCSK9, resulting in increased circulating LDL levels. The increase in LDL could have beneficial effects on host defence by binding and neutralizing LPS and other toxins or by providing a source of cholesterol to macrophages and other cells that play a crucial role in host defence 18. To date, data concerning the effect of human CRP on PCSK9 action is still lacking.

To determine whether CRP induces PCSK9 up‐regulation *in vitro*, we examined the effect of CRP on the protein and mRNA expression of PCSK9 and the underlying pathways in human hepatoma cell line HepG2 cells. Along with testing the primary hypothesis regarding to the role of CRP in PCSK9 regulation, the study was designed to answer the following questions: (1) Is PCSK9 sensitive to the change of CRP concentrations *in vitro*? (2) Does the effect of CRP on PCSK9 affect the physical properties for LDLR degradation? (3) How does CRP modulate PCSK9 expression?

## Materials and methods

### Cell culture and treatment

The human hepatoma cell line HepG2 was purchased from Cell Resource Center, IBMS, CAMS/PUMC. HepG2 cells were cultured in DMEM (Gibco, Grand Island, NY, USA) with 10% foetal bovine serum (FBS) (Gibico), 1% NEAA (Life Technologies, Carlsbad, CA, USA), penicillin and streptomycin at 37°C in an incubator containing humidified air with 5% (v/v) CO_2_, and passaged at 90% confluency with 0.25%(w/v) trypsin‐EDTA. CRP protein was obtained from Sigma‐Aldrich (St. Louis, MO, USA). HepG2 cells were treated with 10 μg/ml of CRP *in vitro*.

### Real‐time polymerase chain reaction (RT‐PCR)

Total ribonucleic acid (RNA) was extracted and reverse‐transcribed (RT) from HepG2 cells. Quantitation of PCSK9 transcript levels was performed by the amplification of cDNA prepared from the isolated RNA with ABI 7500 (Applied Biosystems Inc., Foster City, CA, USA), using the SYBR green PCR master mix (Applied Biosystems Inc.) and primers specific for PCSK9 (forward, 5′‐ATC CAC GCT TCC TGC TGC‐3′; reverse, 5′‐ CAC GGT CAC CTG CTC CTG‐3′, LDLR (forward, 5′‐GAC GTG GCG TGA ACA TCT G‐3′; reverse, 5′‐CTG GCA GGC AAT GCT TTG G‐3′) and glyceraldehydes‐3‐phosphate dehydrogenase (GAPDH) as an internal control (forward, 5′‐GCA AAT TCC ATG GCA CCG T‐3′; reverse, 5′‐ TCG CCC CAC TTG ATT TTG G‐3′) (Takara, Dalian, Liaoning, China). Differences in threshold cycle numbers were used to quantify the relative amount of PCR target contained within each tube. Relative mRNA species expressions were quantitated and expressed as transcript accumulation index (TAI = 2−(▵▵CT)) calculated using the comparative CT method. All values were normalized to the constitutive expression of the housekeeping gene, GAPDH.

### Western blots

Cultured cells were lysed with cell lysis buffer (Beyotime, Shanghai, China). Protein samples were separated by precast NuPAGE Novex 4–12% (w/v) Bis‐Tris gels (Life Technologies) and then transferred onto a nitrocellulose membrane using the iBlotTM dry blotting system as described by the manufacturer (Invitrogen, Carlsbad, CA, USA). Membranes were stained with Ponceau S and blocked in TBST buffer (20 mM Tris, pH 7.5, 150 mM NaCl, 0.1% tween 20) containing 5% non‐fat dry milk for 1 hr at room temperature.

The blots were reacted with primary antibodies overnight at 4°C, then with a secondary antibody conjugated with horseradish peroxidase (HRP) for 2 hrs at room temperature. Blots were developed using chemiluminescence (ECL, Thermo Fisher Scientific,Waltham, MA, USA) on FluorChem M image system.

### DiI‐LDL uptake assay

The LDLR activity of the HepG2 cells was measured. Briefly, the cells were incubated in MEM medium with 5% lipoprotein‐deficient serum (Sigma‐Aldrich) and 20 μg/ml DiI‐LDL (Life Technologies) for 24 hrs at 37°C in the dark. For the fluorescence microscopy, the cells were fixed in the presence of a 4% paraformaldehyde, and the nuclei were subsequently stained with Hoechst dye. Then the cells were examined with fluorescence microcopy (DMI‐4000B, Leica, Buffalo Grove, Illinois, USA.). For the fluorescence quantification, 400 μl of isopropanol was added into each well, and 200μl aliquots were used for the analysis with a infinite M200PRO microplate reader (Tecan, Männedorf, Switzerland, excitation/emission at 530/580 nm). 0.5 mol/l of NaOH was used to lyse the remaining cells and aliquots of 10 μl for protein concentration assay. The ratio of diI‐LDL/protein concentration was used to normalize diI‐LDL uptake value to the cell protein.

### Small interfering ribonucleic acid (siRNA) transfection

PCSK9 Stealth siRNA duplexes (Life Technologies) targeting sequences: 5′‐GAC AUC AUU GGU GCC UCC AGC GAC U‐3′ and 5′‐AGU CGC UGG AGG CAC CAA UGA UGU C‐3′. Hepatocyte nuclear factor 1α (HNF1α) Stealth siRNA duplexes (Life Technologies) targeting sequences: 5′‐UCG AUA CCA CUG GCC UCA ATT‐3′ and 5′‐UUG AGG CCA GUG GUA UCG ATT‐3′. The stealth RNAi negative control Duplex (Life Technologies) were used as a control. RNAi were transfected into HepG2 cells using Lipofectamine TM RNAiMAX (Life Technologies) according to the manufacturer's protocol.

### Chromatin immunoprecipitation (ChIP) assay

The ChIP analysis was performed by using the ChIP assay kit (Active Motif, Carlsbad, CA, USA) according to the manufacturer's instructions. Briefly, the chromatin obtained from HepG2 cells was sheared by sonication, and 1% of the sheared products were collected as an input control. The remaining chromatin preparation was incubated with an anti‐HNF1α antibody or normal mouse IgG (control IgG) at 4°C for 24 hrs. Then, PCR was performed to enrich the promoter binding levels and was carried out using the following PCSK9 ChIP primers: 5′‐TCC AGC CCA GTT AGG ATT TG‐3′(forward) and 5′‐CGG AAA CCT TCT AGG GTG TG‐3′(reverse) 19. The ΔΔCt method was used for the data analysis. Each sample was assayed in duplicate.

### Statistical analysis

Results were expressed as Mean ± S.D. of at least three independent experiments. A value of *P* < 0.01 was considered as highly significant and all values of *P* < 0.05 were considered as significant. All the analyses were performed with SPSS version 19.0 software (SPSS Inc., Chicago, IL, USA).

## Results

### CRP enhanced the expression of PCSK9 but suppressed LDLR

**Figure 1:**
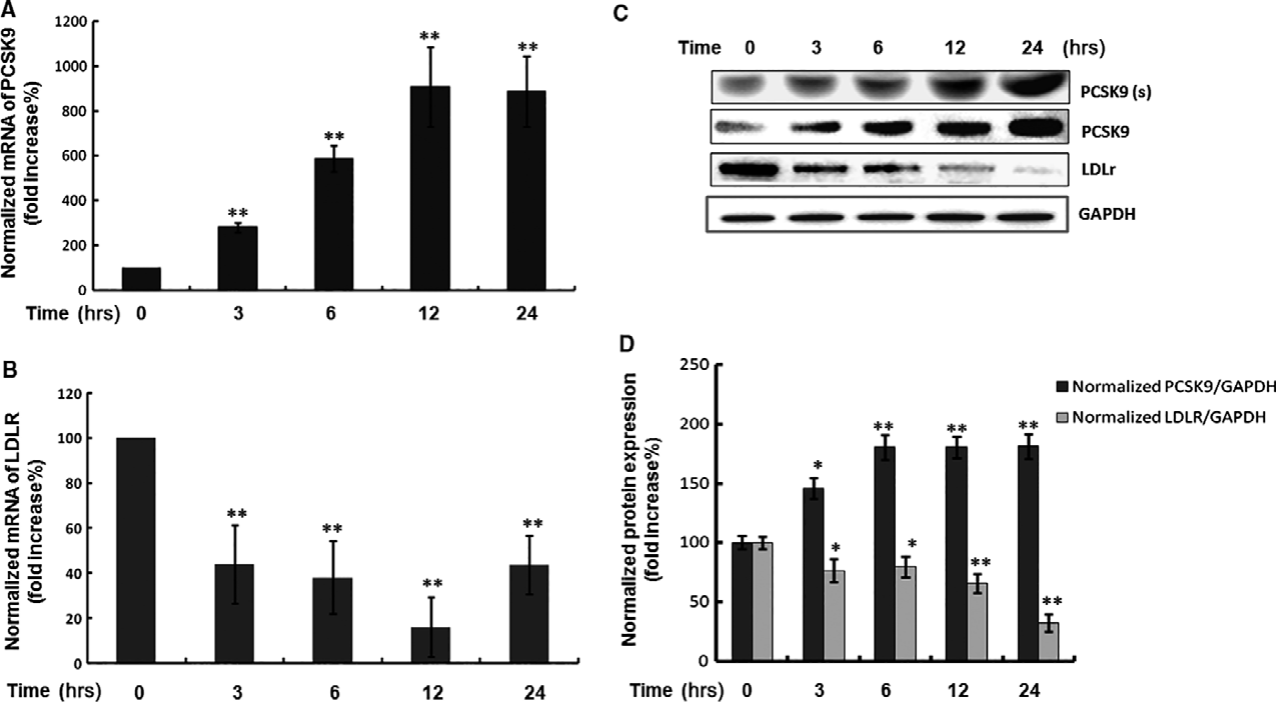
Time course of the protein and mRNA expressions of PCSK9 and LDLR response to CRP treatment in HepG2 cells. (**A**) Real‐time quantification of the PCSK9 mRNA level and (**B**) LDLR mRNA level; (**C**) Western blot analyses of extracellular PCSK9 [PCSK9(s)] and intracellular PCSK9 and LDLR protein levels in HepG2 cells treated with CRP (10 μg/ml) for 0, 3, 6, 12 and 24 hrs. (**D**) The normalized intensity of intracellular PCSK9 *versus* GAPDH was presented as the mean ± SEM of three independent experiments. Significance: *P < 0.05, **P < 0.01 *versus* the 0 hr group. CRP, C‐reactive protein; PCSK9, pro‐protein convertase subtilisin/kexin type 9; LDLR, low‐density lipoprotein receptor.

To clarify the effect of CRP on the expressions of PCSK9 and LDLR, the HepG2 cell serum which was deprived overnight was used, and treated with CRP for different intervals and doses (Fig. S1 A and B). We found that CRP (10 μg/ml) significantly enhanced PCSK9 expression and this dose was used for the time‐dependent investigations. At each time‐point, cell lysates were collected for analysing the messenger ribonucleic acid (mRNA) and the protein expression of PCSK9. Results showed that the mRNA expression of PCSK9 was significantly enhanced by CRP treatment after 3 hrs (*P* < 0.01) and peaked at 12 hrs (*P* < 0.01) (Fig. 1A). By contrast, the mRNA expression of LDLR was decreased significantly by CRP treatment after 3 hrs, and reached the lowest point at 12 hrs (*P* < 0.01), but recovered to the level of 3 hrs at 24 hrs (Fig. 1B). As shown in Figure 1C and D, both the levels of secreted and intracellular protein concentration of PCSK9 were also significantly elevated by CRP treatment while the protein expression of LDLR was decreased in a time‐dependent manner. Moreover, we have observed the protein expressions of PCSK9 and LDLR treated by CRP under the normal serum condition (Fig. S1 C and D). The data showed that the protein expression of PCSK9 was with a high level in the control group, and further increased at 24 hrs. On the contrary, the protein level of LDLR was significantly decreased at 24 hrs.

### The underlying pathways for the PCSK9 regulation by CRP

#### Inhibition of PCSK9 expression by siRNA returned LDLR expression and LDL uptake during CRP treatment in HepG2 cells

**Figure 2:**
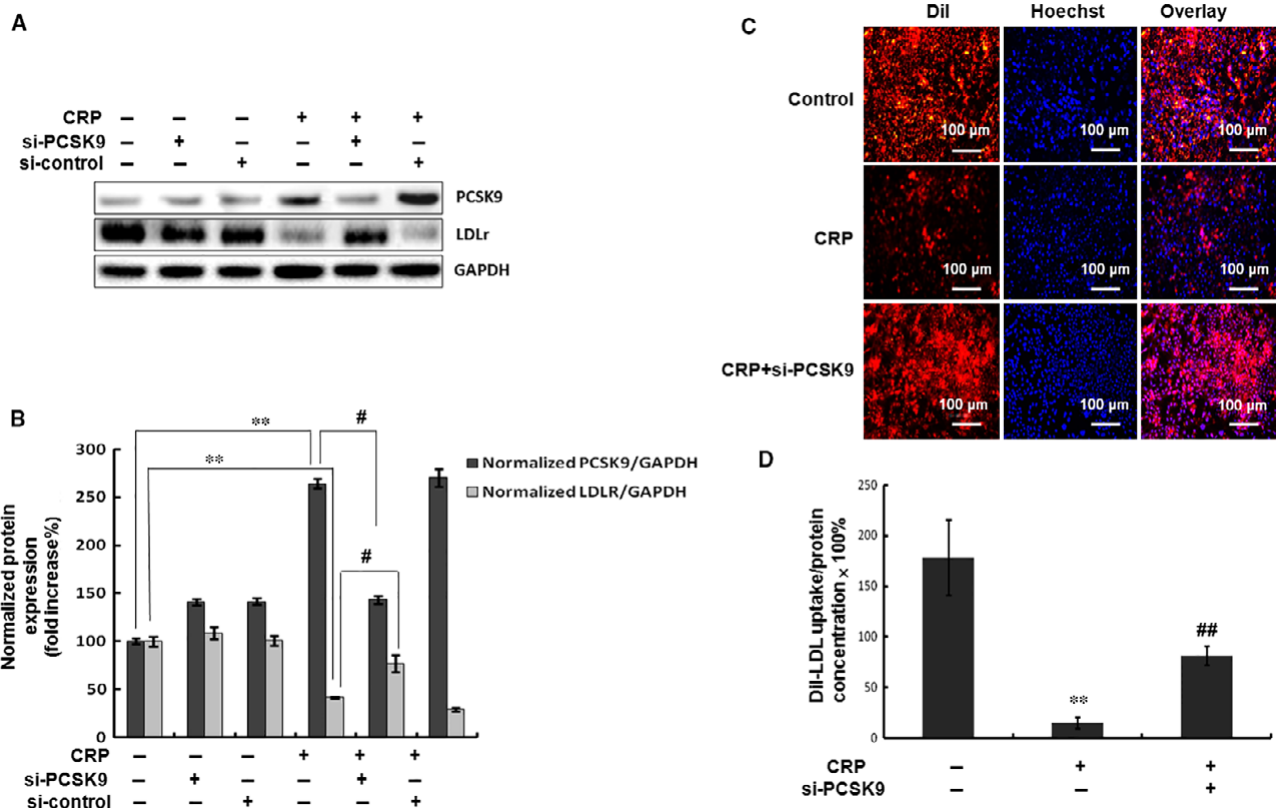
Inhibition of PCSK9 expression returned LDLR expression and LDL uptake during CRP treatment in HepG2 cells. (**A**) (**B**) Western blot analyses of the protein expressions of PCSK9 and LDLR in HepG2 cells transfected with siRNA‐PCSK9 (40 nM) and then treated with CRP (10 μg/ml) for 24 hrs. (**C**) Representative fluorescence microscopy images of cell‐associated Dil‐LDL (red), Hoechst‐stained nuclei (blue) and the overlay. (**D**) Fluorescence of isopropanol‐extracted Dil (520–570 nm, normalized to the cell protein). Values were the representative results of three independent experiments (mean ± SEM). Significance: **P < 0.01 *versus* control; ^#^ *P* < 0.05, ^##^ *P* < 0.01 *versus* the CRP‐treated group. CRP, C‐reactive protein; siPCSK9, small interfering pro‐protein convertase subtilisin/kexin type 9; LDLR, low‐density lipoprotein receptor.

We further pre‐treated the HepG2 cells with the target siRNA to abrogate the expression of endogenous PCSK9, before the administration of CRP protein to investigate the pathway underlying CRP treatment and the subsequent down‐regulation of LDLR was *via* PCSK9. Our results showed that PCSK9 siRNA attenuated the CRP‐induced LDLR degradation in HepG2 cells (Fig. 2A and B). Notably, the LDL uptake of HepG2 cells also presented a similar magnitude of recovery in the reduction after the CRP treatment at 24 hrs (Fig. 2C and D). These data indicated that the PCSK9 could also regulate the degradation of LDLR and LDL uptake accordingly during the CRP treatment in HepG2 cells.

#### Involvement of p38MAPK pathway in CRP‐induced PCSK9 expression and LDL uptake in HepG2 cells

**Figure 3:**
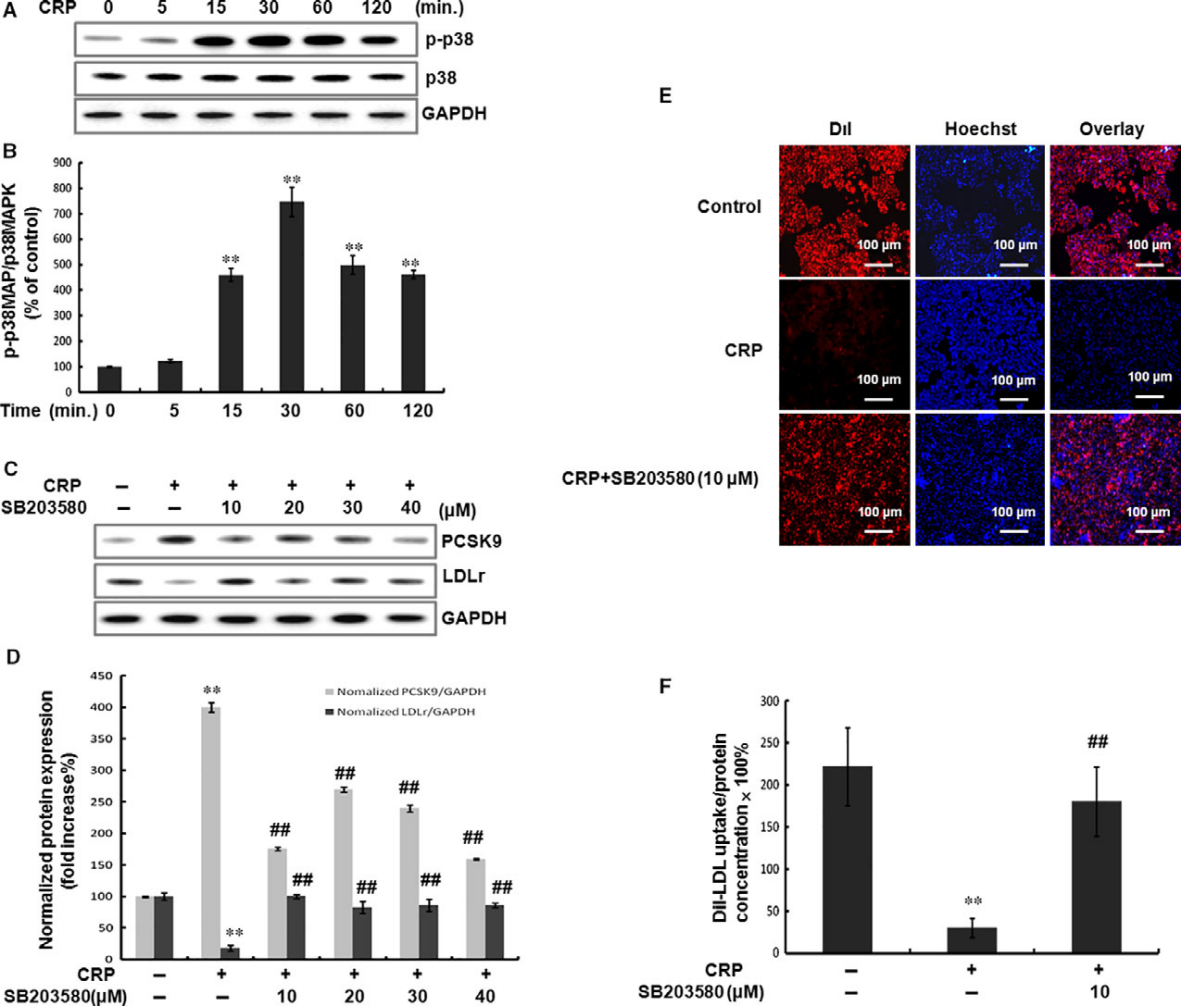
CRP‐induced PCSK9 expression and LDL uptake through p38MAPK pathway in HepG2 cells. (**A**) (**B**) CRP induced activation of signal transduction. Western blot analyses showed the effect of CRP (10 μg/ml) on p38MAPK phosphorylation and p38MAPK expression at 5, 15, 30, 60, 120 min. (**C**) (**D**) CRP induced the up‐regulation of PCSK9 but the down‐regulation of LDLR was inhibited by the p38MAPK inhibitor, SB203580, in HepG2 cells. After serum‐starvation overnight, the cells were pre‐treated with SB203580 (10, 20, 30 and 40 μM) for 1 hr and then stimulated with 10 μg/ml CRP for 24 hrs. The extracted protein samples were analysed by Western blot. (**E**) Representative fluorescence microscopy images of cell‐associated Dil‐LDL (red), Hoechst‐stained nuclei (blue) and the overlay. (**F**) Fluorescence of isopropanol‐extracted Dil (520–570 nm, normalized to the cell protein). Data were presented as mean ± SEM (*n* = 3). Significance: **P < 0.01 *versus* control; ^##^ *P* < 0.01 *versus* the CRP‐treated group. CRP, C‐reactive protein; PCSK9, pro‐protein convertase subtilisin/kexin type 9; LDLR, low‐density lipoprotein receptor.

The mitogen‐activated protein kinase (MAPK) and phosphatidylinositol 3‐kinase (PI3K) signalling cascades have been shown to mediate the action of CRP in different cells 20, 21. To address which of these pathways are in regulated PCSK9 expression by CRP, we used the specific inhibitors, and examined the effects of CRP on the phosphorylations. Results showed that CRP (10 μg/ml) could increase the phosphorylation level of p38MAPK in a time‐dependent manner, and PCSK9 expression by CRP was dependent on the p38MAPK signalling (inhibited by SB203580) (Fig. 3A, B, C and D). However, the total expression level of p38MAPK did not change. By contrast, all other inhibitors of signalling pathways did not significantly affect the expression of PCSK9 with response to CRP (Fig. S2 A–F). DiI‐LDL uptake assay results showed that p38MAPK inhibitor significantly increased the LDL uptake volume (Fig. 3E and F). These data indicated that p38MAPK signalling was involved in regulating PCSK9 and LDLR degradation by CRP in HepG2 cells.

#### Involvement of HNF1α in CRP‐induced PCSK9 expression and LDL uptake in HepG2 cells

**Figure 4:**
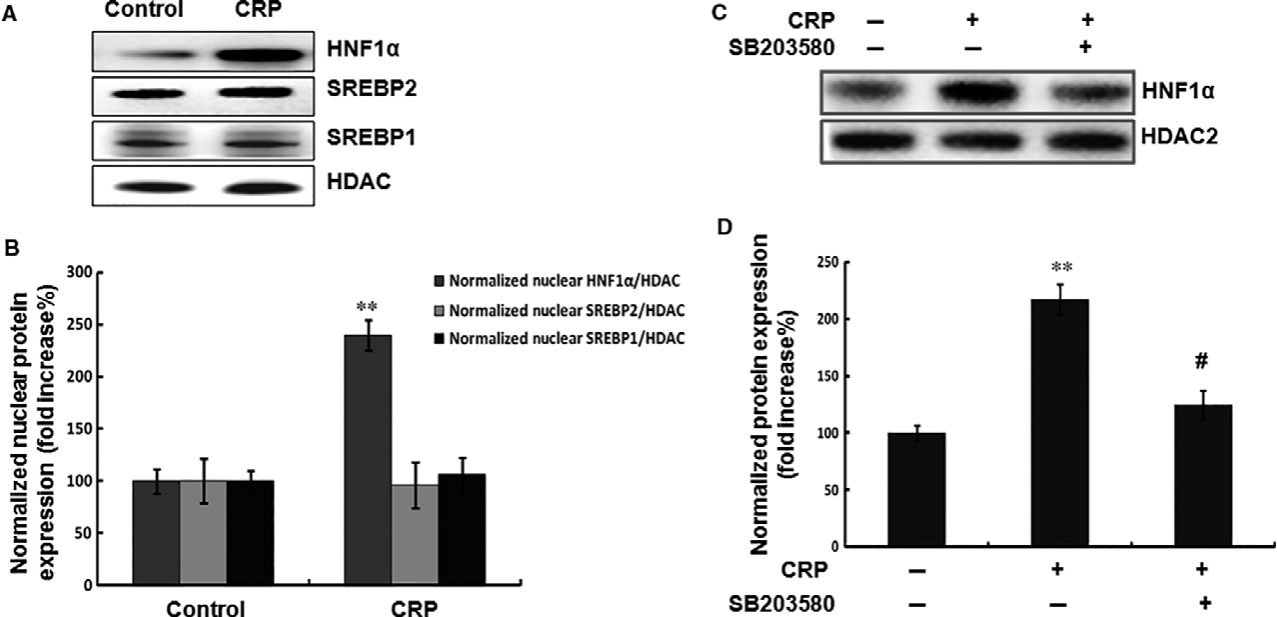
CRP activated the expression of nuclear HNF1α protein but not SREBP2. (**A**) (**B**) Western blot analyses of the transcription factors of PCSK9 including HNF1α and SREBPs in HepG2 cells. (**C**) (**D**) Western blot analyses of the expression of HNF1α after the pre‐treatment with SB203580 (10 μM) for 1 hr and then incubated with CRP (10 μg/ml) for 24 hrs. CRP, C‐reactive protein; PCSK9, pro‐protein convertase subtilisin/kexin type 9; HNF, hepatocyte nuclear factor; SREBP, sterol responsive element‐binding protein.

It has been shown previously that HNF1α and sterol responsive element binding proteins’ (SREBPs) binding sites are the major regulatory elements on the promoter of PCSK9 22, 23, 24, 25. We further investigated whether CRP‐induced PCSK9 regulated by HNF1α and/or SREBPs. The results showed that CRP significantly increased the nuclear HNF1α protein,but not the nuclear SREBP2 and SREBP1 (Fig. 4A and B). To verify whether HNF1α is a critical factor in the p38MAPK pathway, HepG2 cells were pre‐treated with p38MAPK inhibitor (SB203580). The data showed that the levels of CRP‐induced nuclear HNF1α protein were reduced by SB203580 (Fig. 4C and D).

**Figure 5:**
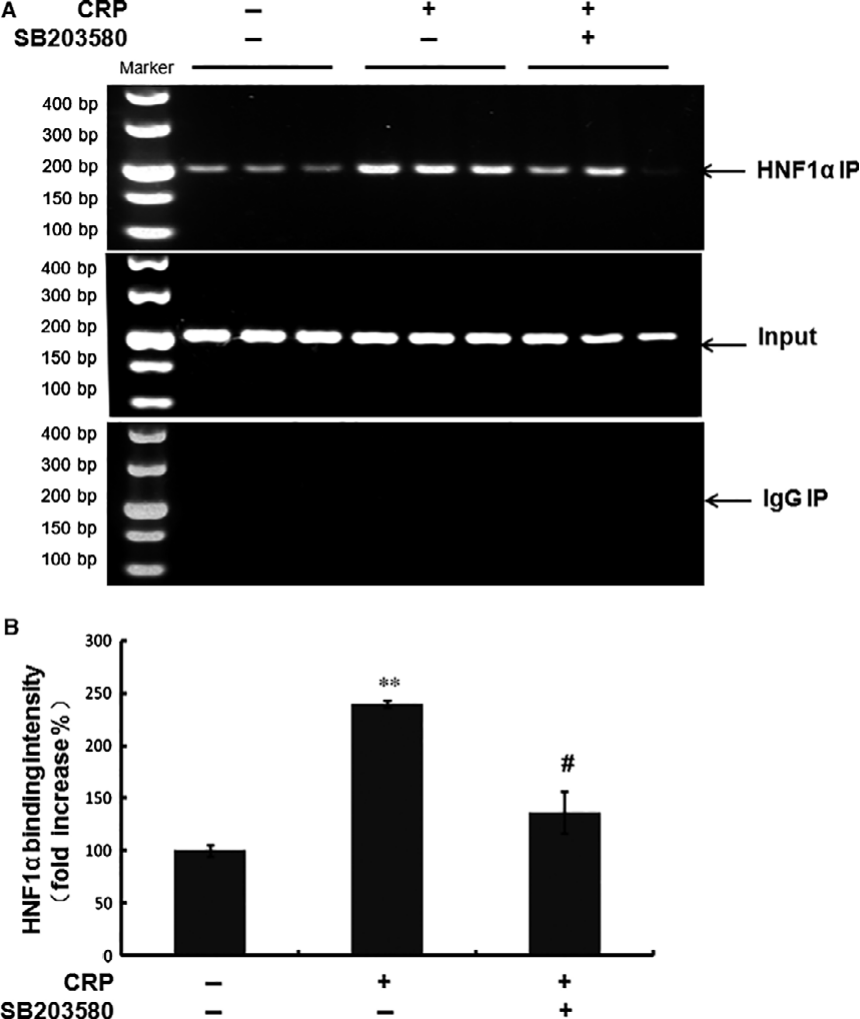
The binding activity of HNF1α to the PCSK9 gene promoter. (**A**) (**B**) Chromatin immunoprecipitation (ChIP) assay of HNF1α binding to the PCSK9 gene promoter. Data were presented as mean ± SEM (*n* = 3). Significance: **P < 0.01 *versus* control; ^#^ *P* < 0.05 *versus* the CRP‐treated group. CRP, C‐reactive protein; PCSK9, pro‐protein convertase subtilisin/kexin type 9; HNF, hepatocyte nuclear factor; SREBP, sterol responsive element‐binding protein.

Furthermore, we examined the binding activity of HNF1α to the promoter of PCSK9 using ChIP assay in HepG2 cells. As shown in Figure 5A and B, stimulation by CRP in HepG2 cells clearly increased HNF1α DNA binding activity. However, p38MAPK inhibitor decreased HNF1α DNA binding activity. These data suggested that HNF1α was involved in p38MAPK pathway leading to CRP‐induced PCSK9 expression.

**Figure 6:**
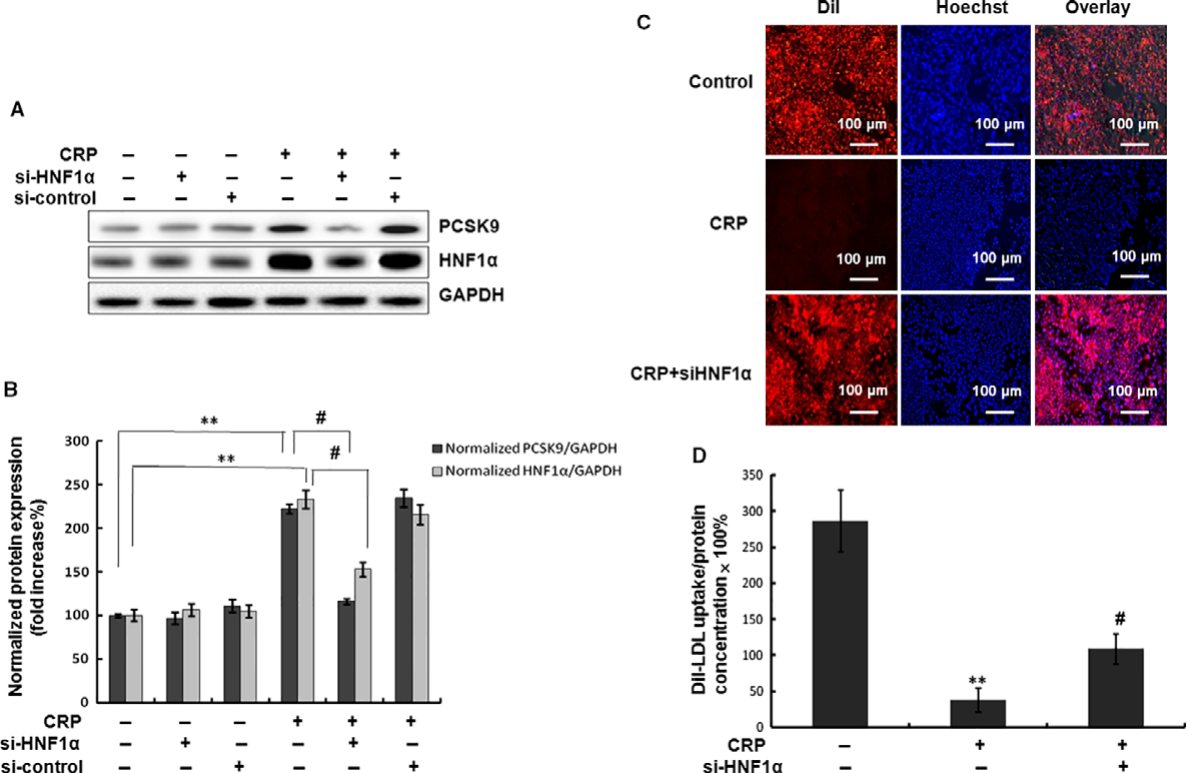
Inhibition of HNF1α attenuated the PCSK9 expression and LDL uptake by CRP in HepG2 cells. (**A**) (**B**) Western blot analyses of HNF1α and PCSK9 in HepG2 cells transfected with siRNA‐HNF1α (80 nM) and then treated with CRP (10 μg/ml) for 24 hrs. (**C**) Representative fluorescence microscopy images of cell‐associated Dil‐LDL (red), Hoechst‐stained nuclei (blue) and the overlay. (**D**) Fluorescence of isopropanol‐extracted Dil (520–570 nm, normalized to the cell protein). Data were presented as mean ± SEM (*n* = 3). Significance: *P < 0.05, **P < 0.01 *versus* control; ^#^ *P* < 0.05, ^##^ *P* < 0.01 *versus* the CRP‐treated group. CRP, C‐reactive protein; PCSK9, pro‐protein convertase subtilisin/kexin type 9; HNF, hepatocyte nuclear factor.

To further confirm that CRP promoted PCSK9 expression by the activation of HNF1α, we transfected HepG2 cells with HNF1α siRNA at the indicated concentration. The results showed that HNF1α and PCSK9 expressions were decreased by HNF1α siRNA (Fig. 6A and B). DiI‐LDL uptake assay data showed that HNF1α siRNA significantly inhibited the suppression of LDL uptake by CRP (Fig. 6C and D). These data suggested that HNF1α was involved in CRP‐induced PCSK9 expression and LDL uptake.

## Discussion

There is controversy as to whether CRP actively contributes to disease progression andconsidered as a true risk factor for certain chronic diseases, or is simply a biomarker of the chronic inflammatory state that accompanies conditions such as dyslipidemia and atherosclerosis 10. Moreover, it is still questionable whether the therapeutic strategies to lower cardiovascular risk with statins should include monitoring CRP as well as cholesterol or not 10, 26, 27. In the present study, we attempted to investigate the challengeable aspect of CRP and provided the first causal linkage of CRP with the expression and function of PCSK9 (Fig. 6), suggesting important new perspectives on the processes by which CRP has a direct negative impact on cholesterol metabolism.

**Figure 7:**
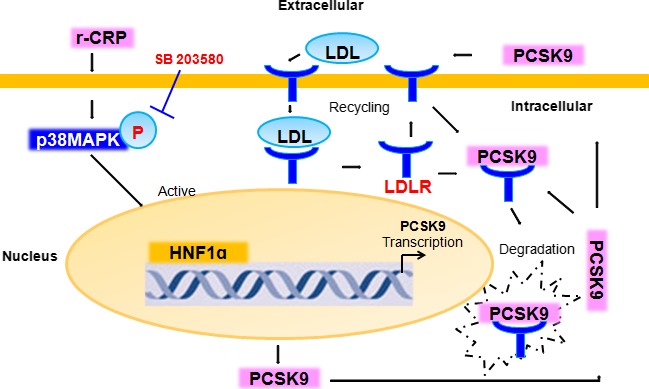
Schematic summary of signal transduction by CRP on PCSK9 expression and function in HepG2 cells. CRP activated p38MAPK pathway, which increased the binding activation and expression of nuclear HNF1α, leading to the PCSK9 expression in HepG2 cells. As a secreted factor, PCSK9 directed the LDLR degradation, preventing the LDLR recycling to the membrane and LDL uptake. CRP, C‐reactive protein; LDL low‐density lipoprotein; LDLR, LDL receptor; PCSK9, pro‐protein convertase subtilisin/kexin type 9; MAPK, mitogen‐activated protein kinase; HNF, hepatocyte nuclear factor.

In fact, the principal findings of the present study were threefold. Firstly, we examined whether the expression or function of PCSK9 could respond to the CRP treatment. As a result, CRP causes a sensitive up‐regulation in PCSK9 but down‐regulation in LDLR. Notably, CRP treatment resulted in a marked decrease in LDLR mRNA levels, which could not be explained by the changes in PCSK9. Therefore, the PCSK9‐independent pathways (or direct effect) of CRP‐induced LDLR decrease might occur, which had been indicated by the previous studies 18, 28. Also, we observed a late return of LDLR mRNA levels at 12 hrs, while the LDLR protein continued to decrease at 24 hrs, suggesting that the late decrease in LDLR protein levels was unlikely to be accounted for the decreases in LDLR mRNA levels while the PCSK9 pathway made sense in the CRP‐induced LDLR protein decrease. Secondly, we therefore explored the effect of PCSK9, which is a key regulator for LDLR protein, during the treatment to investigate the role of PCSK9 in CRP‐induced LDLR decrease. Thirdly, we clarified the signalling pathways to be involved in CRP‐induced PCSK9 regulation in HepG2 cells. Consistently, PCSK9 up‐regulation by CRP in a time‐dependent manner was involved in p38MAPK‐HNF1α pathway. Thus, the possible relationship of CRP with LDLR requires further investigations. But the data of the present study could provide new insights into the role of PCSK9 in CRP‐induced LDLR decrease, and revealed the cell signalling of the pathways (Fig. 7).

Although the investigations on the interaction of CRP with lipid indicating a connection between CRP and atherosclerosis have started from early on in the laboratory 12, no data have been provided yet to link CRP, PCSK9 and LDL uptake. Previously, Zwaka *et al*. 29 isolated monocytes from human blood and transformed into macrophages to explore whether CRP could opsonize native LDL for macrophages. They found that CRP mediated the uptake of native LDL without a need for biochemical modification of LDL *via* CD32, the major CRP receptor on human macrophages, suggesting that CRP binding to LDL in human arterial wall links LDL deposition to the onset of atherosclerosis. Thereafter, Li *et al*. 30 reported the increased effect of CRP on oxidized LDL (oxLDL) receptor (LOX‐1) expression and proposed a new mechanism of CRP‐induced monocyte adhesion and oxLDL uptake *via* LOX‐1 in human aortic endothelial cells. A study from Tits *et al*. 31 directly showed that CRP enhanced the binding of oxLDL to monocytic/macrophage‐like cell *via* Fcγ receptors, influencing the route of oxLDL processing by inhibiting the binding of oxLDL to scavenger receptors. Moreover, Torzewski *et al*. 32 provided *in vivo* data on the atherogenic role of CRP, corroborating the contention that CRP does not play a pathogenetic role in the early atherosclerosis in LDLR−/− mice. These cumulative findings strongly indicated that the atherogenic effect of CRP might depend on its role in cholesterol regulation. In the present study, we investigated the association of CRP, PCSK9 and LDL uptake in HepG2 cells. The possible role of CRP in PCSK9 provided the first direct evidence for causal link between CRP and PCSK9, and identified a novel series of mechanisms by which CRP promoted LDLR degradation but suppressed LDL uptake from the view of PCSK9.

In this study aimed at elucidating the mechanisms by which CRP increases PCSK9, a selective increase in the activity of HNF1α to p38MAPK signalling was demonstrated in HepG2 cells. Previous studies have demonstrated that PCSK9 gene transcription is under the control of SREBPs 22, 23, and the statin treatment depletes the intracellular cholesterol pool, which mobilizes the intracellular proteolytic processing machinery to release SREBP2, which binds to the SRE‐1 element of the PCSK9 promoter and activates transcription. This intrinsic regulatory loop has been recognized as a potentially undesirable limitation to statin therapeutic efficacy in further lowering plasma LDL‐C 5. Recently, investigators have also identified the HNF1α as a pivotal transcription activator for the PCSK9 gene, and the possible involvement of HNF1α in the statin drug action 22. While the exact mechanisms underlying the differential effects of CRP in regulating SREBP2 and HNF1α expression await further investigations. In the present study, we observed that CRP did not activate both SREBP1 and SREBP2 but the HNF1α transcription factor. The potential explanations might be as follows. First, in addition to SREBP regulation, HNF1α, a liver‐enriched transcription factor, is another important transactivation regulator that was found recently to positively regulate PCSK9 expression 33. Second, previous studies including that from Peschel *et al*. reported that curcumin treatment caused an increase up to sevenfold in LDLR mRNA in HepG2 cells, whereas SREBP expression was not significantly changed but the HNF1‐binding element within the PCSK9 promoter was involved in the stimulation of PCSK9 transcription in HepG2 cells 19. Our work might underscore the importance of HNF1α in the control of hepatic PCSK9 expression.

Nevertheless, there were several limitations. First, we suggested that CRP may promote PCSK9 expression and function by affecting HNF1α expression and activity *via* p38MAPK pathway, but the effect of CRP on PCSK9‐related LDL regulations including the mechanisms is far from clear, and more studies, especially mechanism studies, are needed to further clarify. Second, the LDLR protein decrease seemed from both a direct effect of CRP treatment and the effect of CRP on LDLR *via* PCSK9. These two independent effects need further exploration. Third, we only investigated the role of PCSK9 and revealed the cell signalling to show a little story of CRP‐PCSK9‐LDLR. It is of potential interest to extend our present study to explore the interaction of CRP with lipid cholesterol *via* other proteins such as apolipoproteins or other LDL receptors.

In conclusion, these data, for the first time, suggested that CRP could sensitive up‐regulate the expression of PCSK9 inducing a decrease of LDLR and LDL uptake in HepG2 cells through the p38MAPK‐HNF1α mechanism. It is anticipated that further research in this realm will also enhance our basic understanding of PCSK9/CRP associated with cholesterol metabolism.

## Author contributions

Chuan‐Jue Cui and Sha Li completed the project, analysed the data and wrote the manuscript. Li J‐J established the study, interpreted the data and contributed to the reviewed/edited manuscript. The other co‐authors for this manuscript contributed to the experiment. We thank the staff and participants of this study for their important contributions.

## Conflict of interest

The authors have no conflicts of interest to disclose.

## Supporting information


**Fig. S1.** The dose‐dependent effect of CRP on the expressions of PCSK9 and LDLR, and the protein expressions of PCSK9 and LDLR treated by CRP under the normal serum condition. (A) (B) Western blot analyses of extracellular PCSK9 [PCSK9(s)] and intracellular PCSK9 and LDLR protein levels in HepG2 cells treated with CRP (0, 5, 10, 20, 40 μg/ml) for 24 hrs. (C) (D) Western blot analyses of PCSK9, LDLR and SREBP2 during CRP treatment under the normal serum condition. Significance: *P < 0.05, **P < 0.01.

**Fig. S2.** The effects of EPK, JNKI and PI3KI inhibitors on the expressions of PCSK9 and LDLR response to CRP. CRP induced the up‐regulation of PCSK9 but the down‐regulation of LDLR was not affected by the EPK inhibitor, U0126 (A) (B); JNKI inhibitor, SP600125 (C) (D); and PI3KI inhibitor, LY294002 (E) (F) in HepG2 cells. After serum starvation overnight, the cells were pre‐treated with the inhibitors (10, 20 and 40 μM) for 1 hr and then stimulated with 10 μg/ml CRP for 24 hrs. The extracted protein samples were analysed by Western blot. Significance: *P < 0.05, **P < 0.01.
