# Supplementary figures and images for: Enhanced pro‐protein convertase subtilisin/kexin type 9 expression by C‐reactive protein through p38MAPK‐HNF1α pathway in HepG2 cells
Source: J Cell Mol Med. 2016 Sep 15;20(12):2374–83. doi: 10.1111/jcmm.12931 (PMC5134380; doi:10.1111/jcmm.12931)

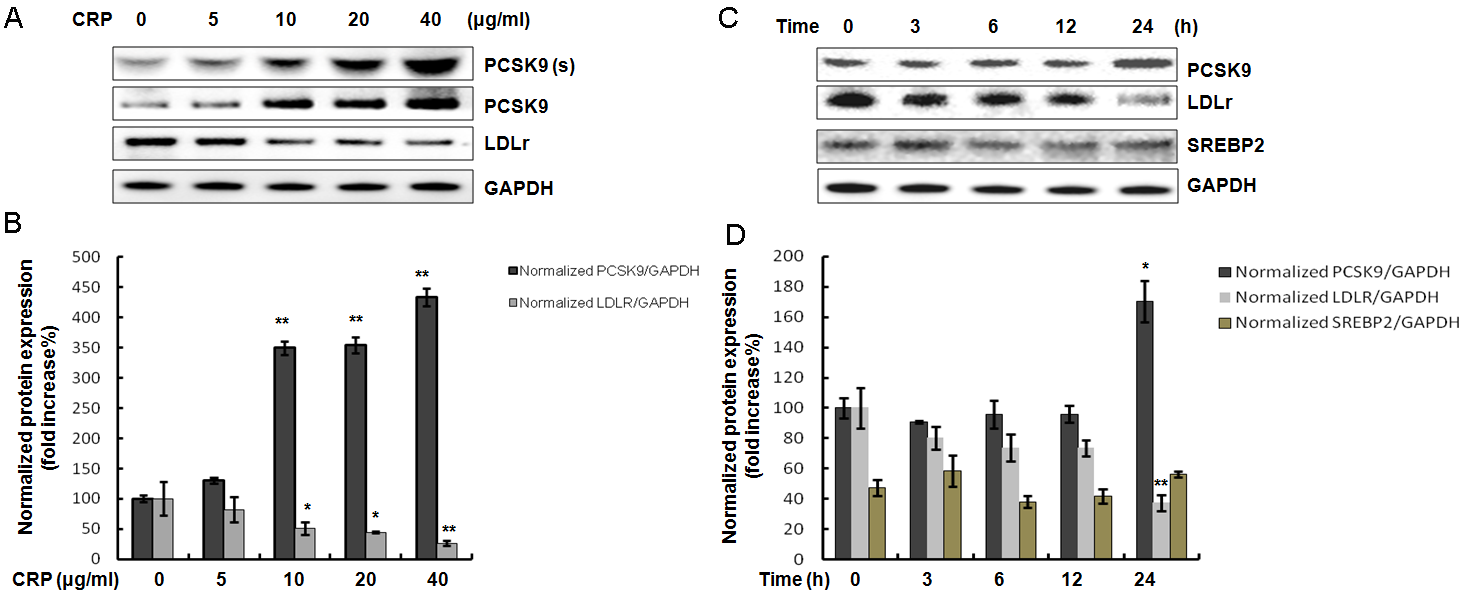

Supplement: Supplementary file 1 — Fig. S1. The dose‐dependent effect of CRP on the expressions of PCSK9 and LDLR, and the protein expressions of PCSK9 and LDLR treated by CRP under the normal serum condition. (A) (B) Western blot analyses of extracellular PCSK9 [PCSK9(s)] and intracellular PCSK9 and LDLR protein levels in HepG2 cells treated with CRP (0, 5, 10, 20, 40 μg/ml) for 24 hrs. (C) (D) Western blot analyses of PCSK9, LDLR and SREBP2 during CRP treatment under the normal serum condition. Significance: *P < 0.05, **P < 0.01. [file JCMM-20-2374-s001.tiff]

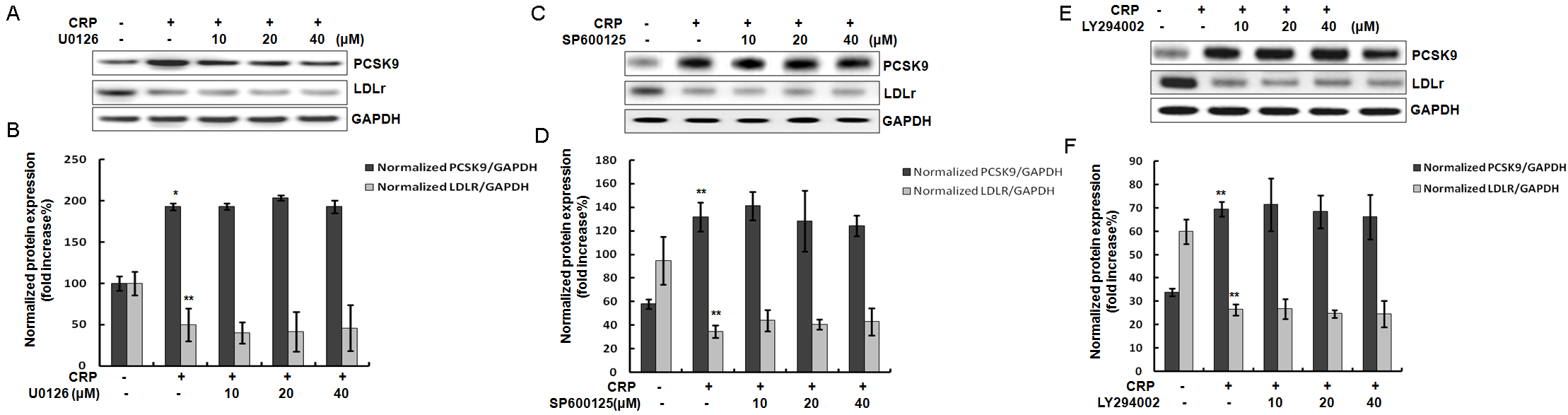

Supplement: Supplementary file 2 — Fig. S2. The effects of EPK, JNKI and PI3KI inhibitors on the expressions of PCSK9 and LDLR response to CRP. CRP induced the up‐regulation of PCSK9 but the down‐regulation of LDLR was not affected by the EPK inhibitor, U0126 (A) (B); JNKI inhibitor, SP600125 (C) (D); and PI3KI inhibitor, LY294002 (E) (F) in HepG2 cells. After serum starvation overnight, the cells were pre‐treated with the inhibitors (10, 20 and 40 μM) for 1 hr and then stimulated with 10 μg/ml CRP for 24 hrs. The extracted protein samples were analysed by Western blot. Significance: *P < 0.05, **P < 0.01. [file JCMM-20-2374-s002.tiff]
