# Supplementary material for: Enhanced pro‐protein convertase subtilisin/kexin type 9 expression by C‐reactive protein through p38MAPK‐HNF1α pathway in HepG2 cells
Source: J Cell Mol Med. 2016 Sep 15;20(12):2374–83. doi: 10.1111/jcmm.12931 (PMC5134380; doi:10.1111/jcmm.12931)
Supplement: Supplementary file 3 [file JCMM-20-2374-s003.docx]

**Supplemental Figures**

**Supplemental Figure 1. The dose-dependent effect of CRP on the expressions of PCSK9 and LDLR, and the protein expressions of PCSK9 and LDLR treated by CRP under the normal serum condition.** (A) (B) Western blot analyses of extracellular PCSK9 [PCSK9(s)] and intracellular PCSK9 and LDLR protein levels in HepG2 cells treated with CRP (0, 5, 10, 20, 40 μg/mL) for 24 hours. (C) (D) Western blot analyses of PCSK9, LDLR, and SREBP2 during CRP treatment under the normal serum condition. Significance: * p<0.05, ** p<0.01.

**Supplemental Figure 2. The effects of EPK, JNKI, and PI3KI inhibitors on the expressions of PCSK9 and LDLR response to CRP.** CRP-induced the up-regulation of PCSK9 but down-regulation LDLR was not affected by the EPK inhibitor, U0126 (A) (B); JNKI inhibitor, SP600125 (C) (D); and PI3KI inhibitor, LY294002 (E) (F) in HepG2 cells. After serum-starvation overnight, the cells were pretreated with the inhibitors (10, 20, and 40 μM) for 1 h and then stimulated with 10 μg/ml CRP for 24 h. The extracted protein samples were analyzed by Western blot. Significance: * p<0.05, ** p<0.01.
